# A qualitative study exploring barriers and facilitators to establishing nurse-led, multidisciplinary psychological care for trauma patients: experiences from doctors and nurses

**DOI:** 10.1186/s12912-022-00971-6

**Published:** 2022-07-19

**Authors:** Yanan Leng, Ying Wu, Zonghua Wang, Xiaoping Zhou, Jianmei Liao

**Affiliations:** 1 Department of Hepatobiliary Surgery, Southwest Hospital, the first affiliated hospital to Army Medical University, Chongqing, China; 2 Department of Clinical Nursing, School of Nursing, Army Medical University, Shapingba District, Gaotanyan Street 30, Chongqing, China; 3 Department of Rehabilitation Medicine, Southwest Hospital, the first affiliated hospital to Army Medical University, Chongqing, China; 4 Department of Nursing, Southwest Hospital, the first affiliated hospital to Army Medical University, Chongqing, China

**Keywords:** Medical staff, Nurses, Trauma care, Qualitative research

## Abstract

**Background:**

Trauma patients are often in a state of psychological stress, experiencing helplessness, sadness, frustration, irritation, avoidance, irritability and other adverse emotions. Doctors and nurses are at the forefront of caring trauma patients and they play a crucial role in psychological supports and mental health care. However, few qualitative studies had based on the framework of the Theory of Planned Behavior (TPB) to explore the experiences in providing psychological care for trauma patients. We examined attitudinal, normative, and control beliefs underpinning medical staffs’ decisions to perform psychological care.

**Method:**

A qualitative study of in-depth semi-structured interviews was conducted among 14 doctors and nurses engaging in trauma care. The participants came from six tertiary hospitals in Chongqing, China. Data analysis was performed using the approach of Colaizzi. According to the framework of TPB, the researchers identified and summarized the themes.

**Results:**

Important advantages (mutual trust, patients' adherence and recovery), disadvantages (workload, short-term ineffective, practice unconfidently), referents (supportive: managers, patients, kinsfolk, nursing culture; unsupportive: some colleagues and patients), barriers (insufficient time or energy, resources situations), and facilitators (access to psychologist, training/education, reminders) were identified. Some demands, such as training diversity, multidisciplinary cooperation and families' support, reflected by doctors and nurses were important for them to carry out psychological care.

**Conclusion:**

According to the TPB, this article explored the internal and external promotion and hindrance factors that affecting the intentions and behaviors of doctors and nurses in implementing psychological care for trauma patients. We also focused on the experience and demands of health professionals in conducting psychological care, which could provide references for managers to formulate corresponding psychological care procedures and norms.

## Introduction

In developing countries, traumatic events are the third leading cause of death with more than 5 million deaths each year, and the rate of disability is exceptionally high [1]. The features of the trauma, for instance, unpredictability, complexity, and uncertain prognosis, might result in patients and their families being unacceptable in the short term. These features could lead to negative psychological outcomes, including fear, anger, resistance, and unassisted, outcomes not conducive to patient's treatment and health care [2]. For example, trauma-related adverse symptoms, such as sleep problems, temper tantrums or nightmares should not continue beyond a few weeks, and 10 ~ 30% of patients developed to posttraumatic stress disorder (PTSD), which could have long-term adverse effects [3]. Persistent posttraumatic pain, risk of infection, economic stress and physical dysfunction are all great strikes to the psychological state of patients. Moreover, Granieri and colleagues [4] surveyed 322 volunteers who had suffered traumatic events, and found that exposure to multiple traumatic experiences might generate severe impairments in self-regulation and identity integration of personality functioning. This could cause patient's psychological distress, which is a common health problem during the first year after injury [5]. Numerous studies have reported that even if the physical trauma of survivors has been cured, psychological trauma can still continue for decades [2, 6, 7]. Psychological problems caused by trauma are significantly associated with generalized anxiety disorder, ineffective coping behaviour, and inactive participation in rehabilitation [2, 8, 9].

Trauma-focused whole-course management should begin in the emergent phase and continue from acute to convalescent phases [10]. Previous studies have demonstrated that psychological care for trauma patients could save more lives, relieve ongoing stress or severe pain, and improve patient outcomes [11, 12]. Individuals' traumatic stress symptoms could be reduced by early multiple session psychological interventions, such as cognitive behaviour therapy, structured writing therapy and internet-based guided self-help [11]. A trauma nurse lead (TNL) program ensures the presence of highly trained trauma nurses to assist other medical staff with continuing care. Research has demonstrated that triage time to diagnostics decreased, and patient and family perceptions of care increased [13]. Zatzick and colleagues [14] established a trauma center based mental health team with a stepped collaborative care intervention, and the results showed that it was associated with modest reduction of PTSD symptom and improved patients' ability to receive social supports. Collaborative and comprehensive processes among medical staff could facilitate interprofessional communication, it might optimize clinical resources, decrease unnecessary and costly admissions, and mitigate medicolegal risk [15]. Moreover, interdisciplinary collaborations and personalized care plans could help patients benefit from the combination of doctors' keen professional judgement and nurses' careful care. Therefore, nurse-lead, multidisciplinary medical staff could provide timely and effective psychological care to cater to the psychological needs of trauma patients.

However, psychological care provided by doctors and nurses to trauma patients still has many problems, such as insufficient implementation strength and poor outcomes. Although nurses have much contact with trauma patients, it is difficult for them to put nurse-led and multidisciplinary psychological care into practice. Few researchers have used a well validated decision-making theory to determine the actual influencing factors and behaviour of medical staff in the process of psychological care. Most current studies have focused on the psychological response of trauma patients rather than paying close attention to the performers. A theory allows health care providers to conceptualize and analyse the processes of carrying out psychological care. Therefore, it is important to explore the facilitators and barriers for doctors and nurses in psychological care based on systematic theory.

The theory of planned behaviour (TPB) originated in social psychology, and includes three variables: an individual’s attitude, subjective norms, and perceived behavioral control, which could be used to understand the elements behind an individual’s intentions to perform behaviour that are under his or her control [16]. Attitude is the first variable of the TPB, and refers to personal beliefs and how they affect individuals’ positive or negative comments about performing a particular behaviour. Subjective norms are the second variable, and they are the social pressures felt by the individual when he or she is considering whether to perform the behaviour. Generally, these pressures originate from the individual's relevant others (e.g., peers, colleagues, families, friends) or groups. Perceived behavioral control is the last variable, and refers to an individual's perception of how difficult or easy it was to perform the behavior. These three variables have a direct effect on behavioral intention, which is reflected the individual's willingness to perform the behaviour [17]. TPB has been widely used to explain the influence of personal, psychological and social factors on the execution of behaviour.

In view of this, the current study aimed to (1) explore the feelings and demands of doctors and nurses in psychological care for trauma patients by qualitative interviews based on the TPB theoretical framework; (2) identify influencing factors of doctors and nurses in implementing psychological care for trauma patients; (3) develop suggestions for constructing nurse-led and multidisciplinary psychological care for trauma patients in the Chinese cultural context.

## Methods

### Design and settings

Capturing a full picture of the potential factors that impact medical staff behaviour in implementing psychological care for trauma patients and offering a reference for managers could help managers develop psychological care norms. We conducted a descriptive qualitative study based on in-depth one-to-one semistructured interviews, which revolved around the themes of behavioral cognition, experience and factors that influence medical staff to provide psychological care for trauma patients.

### Participants

Purposive sampling was used to recruit medical staff who worked in six tertiary hospitals in Chongqing from September to October 2020. The inclusion criteria were as follows: eligible participants (1) had worked in trauma-related fields for at least five years; (2) could think and verbal express themselves well; (3) had participated in psychology-related training or study in medical colleges, work units, other organizations or academic associations and experienced of psychological care for trauma patients; and (4) provided informed consent to participate in this study. The exclusion criteria were follows: participants were ineligible if (1) they have not been on the clinical frontline; (2) they were experiencing major illness; and (3) they were engaging in advanced studies or cycling around different departments in the hospital.

### Data collection

All interviews were conducted on hospital premises in the privacy and comfort of a consulting room belonging to the emergency department and nursing department of Southwest Hospital of Army Medical University in Chongqing, located in Southwest China. Interviews were conducted a time convenient for the participant. The researcher informed the interviewees in detail about the purpose, significance and methods of the study. Consented participants underwent semistructured in-depth one-to-one interviews face-to-face or on the telephone. The interview questions according to TPB guidelines revolved around messages about behavioural beliefs concerning the advantages and disadvantages of psychological care; normative beliefs about those who would support and oppose psychological care; and control beliefs comprising barriers and facilitators related to performing psychological care. The interview outline was initially developed by WZH (health care researcher with an education and a work background in nursing, trauma and psychology). Then, questions were piloted tested by two nurses and one doctor. The formal interview process was conducted by one trained interviewer (LJM) to ask questions according to the interview outline, and the other two researchers (LYN and ZXP) were responsible for observing and recording the interviewees' verbal and nonverbal expression. Each interview was audio-taped and transcribed verbatim, and lasted approximately 30 to 45 min. Repeated data became to emerge and any new information was obtained, which indicated the data saturation, and the interview could be ended [18, 19].

Some examples of specific questions are listed as follows: (1) What is your attitude towards performing psychological care for trauma patients? (2) What are some advantages or disadvantages of implementing psychological care for trauma patients? (3) Who are the people (or groups of people) influence on you who would support you in implementing psychological care? (4) Who are the people (or groups of people) influence on you who would not support you in implementing psychological care? (5) What are the barriers and facilitators to establishing psychological care for trauma patients? (6) What is needed to promote the effective implementation of psychological care?

### Data analysis

Two researchers (LYN and ZXP) involved in the interview immediately sorted and transcribed the digital recording data, and verified transcripts with the field notes to ensure the integrity and authenticity of the data. In this study, Colaizzi's 7-step analyse method was used to analyze the interview data [20]: we read all the interview data in detail; extracted meaningful statements; coded recurring views; analysed the meaning of the common form theme; elaborated completely on the topic in relation to research phenomena; identified similar views; and returned to the interviewee for verification when there was doubt. Two researchers familiar with the subject matter (WY and WZH) read and considered all the collated extracts for each theme to ensure that they formed a coherent pattern. Colaizzi's 7-step analyze deductive inductive strategy is the content analysis method for summarizing and interpreting meaningful exposition from data. Theory makes sense of social phenomena and provides a specific focus to different aspects of research data and a framework to conduct an analysis [21]. The TPB offered the analytic coding process and provided a framework for the identification and coding that potentially included data relating to the study aims and theoretical constructs [22]. Based on the perspective of TPB theory, we embedded the summarized themes by means of the Colaizzi's 7-step analyze method into the TPB theoretical framework, which helps us to comprehensively and systematically identify and summarize the factors affecting the behavior and behavior intention of medical staff in the implementation of psychological care. Examples of the data analysis are presented in Table 1. Manual coding and classification of data was adopted. All procedures were conducted in accordance with the Declaration of Helsinki.

**Table 1:** Examples of the data analysis

Data extract	Code	Sub-theme	Theme
1. Psychological care could be reflected in the full exchange and communication with patients, then it was conducive to establish a good relationship with patients	Forming mutual trust	Behaviour beliefs in – Advantages	Attitude
2. The patient was able to follow the instructions of the medical staff, such as positioning and taking medication regularly	Adherence to therapy		
3. After psychological counselling, patients could face the condition well, cooperate with treatment and nursing, and promote they return to society as soon as possible after recovery	Recovery promotion		
4. Everyone (medical staff) was busy. Offering psychological care would increase the workload	Increase workload	Behaviour beliefs in – Disadvantages	
5. The exact effect of psychological care might not be seen immediately, so it would not be done as detailed as the beginning	Short-term ineffective		
6. We could do not much. It stayed at the level of health education, functional guidance and medical information. One-sided verbal comfort was the most common	Unconfident to practice		

### Rigor

The accuracy of the transcripts was verified by two authors (LYN and ZXP) through a process of concurrently reading and listening to the recorded interviews. Pre-interviews were piloted tested by two nurses and one doctor to make sure the interview outline rational and the objects representative. The transcribed materials were reviewed by two researchers (WY and WZH) with rich clinical practice and qualitative research experience, and then two researchers (LYN and ZXP) who participated in the interview process independently analyzed the verbal and non-verbal information and determined the themes through group discussion to improve the reliability. To confirm conformability, investigator triangulation was used. The interviewees did not communicate with each other, which also ensured the process rigor.

## Results

### Participant characteristics

The sample size followed the principle of information saturation. Finally, fourteen participants were recruited from September to October 2020, including ten nurses (No. N1 ~ N10) and four doctors (No. D1 ~ D4). Participants ranged from 31 to 51 years old (mean age of 39.36 years), and they had worked in trauma-related fields for a duration of 15.57 years (range: 5 years to 33 years). The detailed characteristics of the participants are shown in Table 2.

**Table 2:** Characteristics of the participants (*n* = 14)

No	Gender	Age (years)	Working years	Years of work in the trauma field	Department	Educational background	Professional title
D1	Male	44	20	15	orthopedics	undergraduate	visiting staff
D2	Male	35	8	8	traumatic burn department	master	visiting staff
D3	Male	48	24	24	orthopedics	doctor	visiting staff
D4	Male	34	8	5	emergency trauma department	undergraduate	visiting staff
N1	Female	39	20	10	emergency trauma department	undergraduate	associate professor of nursing/head nurse
N2	Female	38	15	5	emergency trauma department	undergraduate	intermediate nurse
N3	Female	51	33	33	orthopedics	undergraduate	professor/head nurse
N4	Female	38	19	19	traumatology department	undergraduate	intermediate nurse
N5	Female	37	18	18	traumatology department	undergraduate	intermediate nurse
N6	Female	31	17	13	orthopedics	undergraduate	intermediate/head nurse
N7	Female	40	17	17	emergency department	undergraduate	intermediate/head nurse
N8	Female	43	23	23	orthopedics	junior college	intermediate nurse
N9	Female	39	16	16	orthopedics	undergraduate	intermediate nurse
N10	Male	34	12	12	emergency department	undergraduate	intermediate nurse

## Themes

**Fig. 1:**
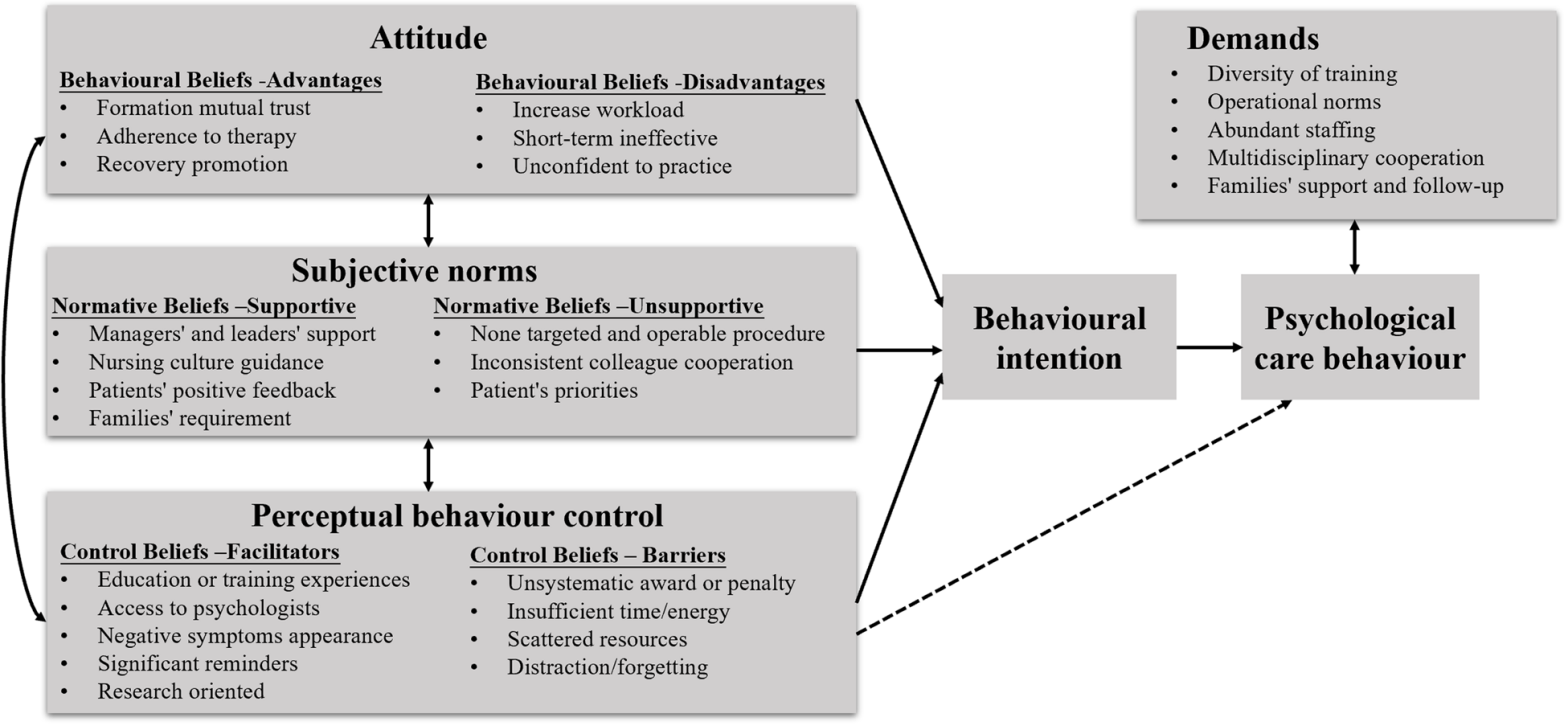
Domains of psychological care behaviour among medical staff based on the TPB

Four main themes concerning psychological care for patients by medical staff were identified based on the data and TPB: attitude, subjective norms, perceptual behaviour control and demands. The domains of the four themes are presented in detail in Fig. 1. Considering the settings and context for our practice, we modified the definition of the three determinants from the theory of planned behavior. Attitude in this study refer to beliefs and evaluations of the advantages and disadvantages providing psychological care for trauma patients. Subjective norms reflect the supportive or unsupportive perceptions felt by medical staff from hospital employers and family members, which may influence whether or not they perform psychological care for trauma patients. Perceptual behavioral control refers to the medical staff's perception of barriers and facilitators in carrying out psychological care for trauma patients. Demands refer to the suggestions and needs of medical staff in the process of carrying out psychological care.

### Theme 1: Attitude

Most clinical nurses attached importance to psychological care and took the initiative to provide basic and broad psychological care for trauma patients in clinical practice. This was beneficial for the patients to relieve stress, improve cooperation and compliance with treatment, promote recovery, and gain mutual trust. However, they also mentioned that psychological care required more related professional knowledge and competencies, which led to increased workload. Moreover, the psychological care might not take effect immediately, which might affect the passion of the implementers.

#### Beliefs in advantages

After receiving psychological care, trauma patients might relieve their anxiety, so that they could treat diagnosis and treatment objectively and positively. This process made medical staff feel professional identity and sense of achievement. Under the control of this belief, medical staff paid special attention to whether psychological care could bring potential benefits to patients, such as forming mutual trust, adhering to treatment and promoting recovery.

Some interviewees said that appropriate and timely psychological care by medical staff could help patients find their real problems and needs, assist them with role change, and establish mutual trust.

N2: "It was important to establish trust with the patients. We could provide a comfortable environment, gentle operation, friendly language, and use video news and other media to divert patients' fretful attention."

N6: "Psychological care could be reflected in the full exchange and communication with patients, then it was conducive to establish a good relationship with patients."

Most interviewees mentioned that psychological care could let patients correctly cope with the impact of the trauma and improve their treatment compliance.

D2: "Some trauma patients undergo great pressure and have difficulty falling asleep before surgery. Through the verbal communication of doctors, the information support provided by nurses and the company of family members, they could gradually fall asleep."

D4: "The patient was able to follow the instructions of the medical staff, such as positioning and taking medication regularly."

Receiving psychological care could alleviate patients' negative emotions, reduce pressure, increase confidence in treatment, and promote their successfully return to society.

N7: "After psychological counselling, patients could face the condition well, cooperate with treatment and nursing, and promote they return to society as soon as possible after recovery.

N9: "Patients were less anxious and cooperated actively with our treatment. Sleep, diet and metabolism recovered quickly, which might help shorten the treatment process."

#### Beliefs in disadvantages

Potential disadvantages contribute to negative attitudes among participants, including increasing workload, reflecting into short-term ineffective psychological care, and practising unconfidently.

More than half of the interviewees believed that trauma occurred suddenly and urgently, which would cause great physical and mental impacts on patients. Trauma patients might depend strongly on medical staff and families, which requires medical staff to devote more energy and attention to them.

N5: "After injury, the patient strongly wished for his family to be around him and longed for the meticulous care of medical staff."

D2: "Everyone (medical staff) was busy. Offering psychological care would increase the workload."

A few interviewees mentioned that the psychological care process might be time-consuming and laborious, and could not be effective in the short-term.

D2: " The exact effect of psychological care might not be seen immediately, so it would not be done as detailed as the beginning."

Due to insufficient psychological care-related knowledge, the skill of carrying out psychological care was still relatively singular. Half of the interviewees mentioned that they had no ability to implement comprehensive, in-depth, systematic and personalized psychological care for trauma patients.

N5: "We could do not much. It stayed at the level of health education, functional guidance and medical information. One-sided verbal comfort was the most common."

*N6: "The clinical experience and educational level of nurses was uneven. Most nurses with psychological care qualifications were concentrated in the psychiatric department. The psychological care professional degree of nurses in the trauma ward was generally not high."*

### Theme 2: Subjective norms

Subjective norms originated from a person's beliefs about whether important referents approve or disapprove of them carrying out the behaviour (normative beliefs, e.g., other medical staff would support me performing psychological care).

#### Normative beliefs -supportive

The medical staff considered recognition by leaders and managers to be the most salient referents supportive of their performing psychological care. Other supportive referents were nursing culture guidance, patient's positive feedback, and the requirement of the families.

At present, the implementation of psychological care for trauma patients has gradually been valued and advocated by hospital managers. Eleven interviewees said that leaders carried out physical and mental care for trauma patients in the form of organizing expert lectures, building up volunteer service teams, setting up multidisciplinary collaboration teams, and conducting surveys on patients' psychological conditions. This also encouraged interviewees to take the initiative to carry out psychological care.

N5: "Our hospital launched the physical and mental care case sharing contest, which contributed to establishing a humanistic care group aiming to mutually exchange of psychological care methods."

Nursing culture advocates the implementation of physical and mental holistic care for patients and fully embodies humanistic care. Sympathy and responsibility for the patients are rooted in the medical staff. Several interviewees believed that driven by this nursing culture and sense of responsibility, they would take the initiative to care for patients' psychological conditions.

N3: "The basic moral character and duty of the medical staff was to relieve the pain of the patients, provide care and companionship, which was obligatory."

A few interviewees expressed that the positive affirmation and feedback of trauma patients on psychological care and the establishment of an interactive relationship between medical staff and patients would make the implementers feel professional value and sense of achievement, and motivate them to carry out psychological care.

N5: "After communication, patients took the initiative to greet us and offered us the feelings feedback on the process of care, we would feel a sense of achievement."

N8: "Psychological problems of patients could be relieved, they realized that nurses did not just give injections and infusion, we also embodied our professional values."

A minority of respondents reported that family attention to the psychological status of trauma patients would also determine the behaviour norms of medical staff.

N1: "It was an urgent need for family to help patients establish confidence in recovery when they encounter trauma."

#### Normative beliefs -unsupportive

The medical staff believed that not having none targeted and operable procedures by leaders and managers to be the most salient referents unsupportive of performing psychological care. Participants identified inconsistent cooperation originating from colleagues and patient's priorities to receive other support rather than psychological care as unsupportive factors.

All interviewees indicated that managers had not yet formulated targeted and operable procedures and norms, so medical staff could not take timely assessment and reasonable treatment when facing the psychological conditions of trauma patients, which greatly limited the effective implementation of psychological care.

N6: "There was no professional assessment of the effectiveness of psychological care, and there was also no clear specification of what psychological care contains."

D4: "We did not have specific psychological care measures, so we did not know whether to comfort or persuade patients."

The implementation of psychological care was a long process, and inconsistent cooperation between colleagues for the same trauma patient would affect its development.

N2: " There was a lack of consistency in psychological care, and the successor nurse did not know the extent and specific content of the shift nurse's implementation of psychological care for patients."

Most interviewees mentioned that some trauma patients would reject and resist sudden changes in the environment and treatment process, and they would show emotions such as rejection, unacceptability, self-denial, and fear. Some interviewees believed that especially in the posttraumatic period, patients might be more concerned about the impact of trauma on their physical function, social survival, and later rehabilitation. Psychological care was not the patient's primary need, which made medical staff lack motivation to implement it.

N5: "When patients suddenly injured, they would deny and resist at first, avoid recalling the injury scene."

N8: "The patient most worried about his trauma condition. Sometimes when you were doing psychological care, he was not willing to talk with you. He felt that you hadn't really mentioned what he wanted to know."

### Theme 3: Perceptual behaviour control

#### Control beliefs -facilitators

Participants noted that education or training experiences encouraged psychological care. When trauma patients exhibited psychiatric and psychological symptoms, their particular psychological condition was reflected in the nursing records of nurses, and the medical records of doctors, as a prompt reminder, was considered a motivator to encourage psychological care. Offering trauma patients access to professional psychologists was also one of the facilitators to benefit psychological care. In addition, participants noted that psychological care was, as one of the researches focuses, positively oriented in performing psychological care.

The majority of interviewees affirmed that professional and systematic psychological knowledge and skills training has a certain role in promoting the implementation of psychological care. This experience helped performers exchange ideas, master certain psychological care methods, and facilitate the provision of targeted care for patients. In addition, half of the interviewees said they had been exposed to psychology-related courses at school, which could potentially lead them to consider patients' psychological conditions in their clinical practice.

N7: "Inviting professional psychological trainers to carry out lectures could promote medical staff's understanding and experience of psychological care, which was conducive to its better application in clinical practice in the future."

D1: "Through learning some psychology-related courses in the school, medical staff had a certain understanding of psychological care. Afterwards, they would have the awareness to implement psychological care when the patients might need it."

The psychological characteristics and requirements of different trauma patients varied greatly, which would cause confusion to implementers. Most interviewees believed that psychological care might be more effective when it could be conducted by psychology professionals.

N9: "After simple screening with the scale, patients with psychological problems would receive guidance from the psychologist and pharmacist."

D1: "With these patients who had some psychological problems or obstacles, we generally sought professional psychologists for consultation and guidance."

Some interviewees mentioned that trauma easily caused physical and mental harm to patients, and when patients were in negative emotional states or behaviours, it promoted medical staff to pay more attention to patients' psychological conditions.

N7: "Patients might express delusions, hallucinations and suicidal tendencies when they experienced too severe traumatic events or scenes, which would prompt us to strengthen the judgement and observation of patients' physical and mental conditions."

N10: "Young people or unmarried people with an impaired physical image might experience mood disorders. They could be at risk for depression or suicide and should be closely cared for by medical staff."

Most interviewees pointed out that the abnormal psychological condition of patients and the counselling and disposal by medical staff would be noted in the nursing records of nurses and medical records of doctors, or the special situation might be highlighted during a shift meeting, which could remind the medical staff to improve the patient's bad psychological condition.

N8: "If the patient had a special psychological condition, we would describe it in the nursing records."

N9: "We would reflect the psychological status of patients in the nursing records, and paid attention to the effect of psychological counselling."

Some interviewees mentioned that psychological care was one of the focuses for researchers. Medical staff could deepen their cognition of psychological care, stimulate interest and apply the research output to clinical practice by exploring related research.

N1: "From the past to now, psychological care had always been mentioned. It was of great significance for nurses to carry out psychological research."

#### Control beliefs – barriers

Unsystematic award or penalty mechanisms were discussed by the medical staff as barriers to performing psychological care. Other frequently nominated barriers included insufficient time or energy and scattered recourses of understanding psychological care. Distraction and forgetting were also mentioned as barriers.

Almost half of interviewees mentioned that missing standardized supervision and reward mechanisms at the management level would cause psychological care performers to gradually lose enthusiasm, which could hinder the effective development of psychological care.

N6: "The implementation of psychological care was more like the duty of nurses, without practical incentives, nurses would gradually lose the enthusiasm to carry out it."

D1: "We were encouraged to do psychological care, but there was no compensation for this project, and there was also no evaluation index after the implementation, which would affect the enthusiasm of the implementers."

Clinical practice is complicated and busy. All the interviewees showed that the lack of human resources greatly restricted the continuous and detailed development of psychological care. Medical staff might have insufficient time or energy to perform psychological care after ensuring that their routine work was completed.

N6: "Nurses did not have leisure time to get out of their trivial routine work."

The majority of interviewees believed that the relevant knowledge and skills acquired through school courses or intensive psychology-related learning organized by the hospital were scattered. The training time was usually fragmented (such as several times a week), and the theoretical knowledge was not closely connected with the practical application. Therefore, these scattered resources made it difficult for performers to truly master the psychological care related-knowledge and apply for clinical practices.

N2: "Infrequent systematic training might be organized once or twice a year indeed, the impression after learning was not deep."

N5: "It was difficult for medical staff to get systematic understanding and clinical practice application by arranged several trainings of psychological care in scattered time."

Only one interviewee mentioned that nursing staff would arrange the priority of tasks according to the nursing plan. When patients did not show significant psychological problems and their daily tasks were heavy, the implementation of psychological care might be deprioritised or forgotten.

N6: "There was many basic jobs to be done for medical staff. When the implementation of psychological care was not the primary task for them, this work was easy to ignored."

### Theme 4: Demands

The following demands were desired to be supported for medical staff: diversification of training, establishment of operational norms, ensuring abundant staffing, promotion of psychology-related multidisciplinary cooperation and cooperation with family members.

The interviewees thought that diversified training forms and contents were the basis and key to implementing psychological care. Many interviewees said that operability, normative and targeted psychological care procedures and norms should be established.

N3: "It was necessary to clarify the scope of application of the norms and consider whether different levels of hospitals and nurses could accept or not. "

N6: "The offline and online training methods could be carried out simultaneously, which was convenient for later medical staff to flexibly arrange time for review."

Most interviewees suggested that adequate and reasonable staffing should be provided.

N6: "Advocating or encouraging medical staff to offer psychological care to patients should be combined with the current manpower."

Overwhelming majority of interviewees suggested that systematic and targeted psychological care required high professional skills, and multidisciplinary team assistance could be considered when patients had significant psychological problems and significant needs.

N3: "If the nurse did not have abundant psychological knowledge and skills after the problem was found, she should learn to play the team advantage."

A few interviewees mentioned that psychological care needs to mobilize the patient's support system and pay attention to continuity.

N1: "The support of families to patients could not be replaced by medical staff. Psychological care should not only stay in the hospital, but it should continue after discharge."

## Discussion

The interviewees believed that the current psychological care for trauma patients has a single form and insufficient connotation, and fails to fully combine the different trauma characteristics and periods. Psychological care was dominated by nurses, which was often expressed through comfort words, nonverbal physical contact (such as hugging, massage, etc.) and health mission in daily work. Medical staff did not know much about the contents and methods of professional psychological care, and due to heavy routine work, they were not positive enough to mobilize patients to participate in the implementation of psychological care decision-making and the whole process. Attitude will affect the emotional tendency towards things, and ultimately affect behaviour. Different nurses had various clinical experiences, educational levels, and education or training levels in psychological care, which would affect their attitudes in carrying out nursing activities [23]. Sijbrandij and colleagues [24] trained 63 primary health care workers in psychological first aid (PFA). They used participatory learning such as role-play. Then, this enhanced the knowledge and ability of help preparation, situational observation, listening, referral, and self-care, and effectively improved their knowledge and understanding of social psychological support strategies, roles and responsibilities, and professional attitudes, and the effect still existed six months after the training. Therefore, managers should consider the characteristics of trauma patients, utilize the forms of workshops, situational simulation, case analysis and the focus of discussion, and combine the flexible methods of online or offline organizations to strengthen professional training and guidance for medical personnel in the domain of trauma to allow medical staff to fully recognize and pay attention to this behaviour. Managers should also recognize that some training effects might only be evident during follow-up rather than immediately after training, so providing opportunities for medical staff to put training into practice was key to consolidating training knowledge and skills.

Majority of the participants approved various forms of hospital support for the behaviour. In the face of the patient's trauma, the families usually have deep psychological injuries that are helplessness and regret, and they are eager for professional medical staff to provide timely counselling for patients and be informed of their physical and mental conditions. In fact, when nurses encounter trauma patients and their families, they might feel overwhelmed, frightened and helpless, which should be due to the inability of nurses to provide timely medical services that patients need in most cases [25]. It is suggested that managers should guide trauma nurses in a timely manner to enhance moral courage and resilience to cope with adverse emotional conditions when dealing with trauma patients. Meanwhile, medical staff should provide timely feedback on the problems, suggestions and feelings to managers and patients' families in the process of carrying out psychological care, so that they can fully understand the process, content and significance of psychological care, which could also help the implementers gain deeper support.

The more experiences and resources medical staff had (such as receiving psychological education and training experiences, having access to psychologist to assist patients in counselling and getting some significant reminders, etc.), the more confident they were in their own abilities and the more likely they were to initiate behavioral intention. Some hindrance factors (such as insufficient time or energy, lack of psychological care implementation mechanisms, difficult cooperation of patients and their families, etc.) would increase the difficulty of implementing the behaviour. When medical staff had the more information and resources, the easier it was to be make use of, the less resistance they would face, and the more likely they would actually perform this behaviour. It was suggested that leaders fully integrated information and resources, provided diverse psychological care intervention programs at home and abroad for performers, optimized the process of implementation of infrastructure conditions, encouraged multidisciplinary professionals to assist and guide, participated in the patient's psychological care implementation decisions, and paid attention to psychological screening, assessment, treatment, feedback and follow-up in the process of labour division and cooperation.

According to the in-depth interview results, trauma patients had strong dependence on medical staff and obvious psychological care needs, which to some extent aroused the subjective will of medical staff to carry out psychological care, and then promoted their psychological care behaviour. A qualitative study from the UK National Health Service showed that trauma patients indicated the value of being understood, having their emotional needs recognized and addressed, quality of interaction with providers, empathetic care that extended beyond medical needs, coordination of care, and positivity of care delivery as important dimensions of quality care with implications for their recovery [26]. However, due to the complicated daily work of medical staff, the lack of professional knowledge and skills related to psychology, and the unobvious effect of psychological care, the overall enthusiasm towards psychological care for trauma patients was insufficient. Therefore, it is suggested that medical staff should pay more attention to patients with severe psychological distress caused by trauma, actively build a platform for information sharing and collaboration between medical staff and patients, open and transparent mechanisms of division of labour and cooperation between psychological care teams, consult patients' opinions, effectively improve the effectiveness of psychological care, and further stimulate the willingness of medical staff to implement.

Finally, the majority of interviewees indicated the current lack of psychological care procedures and norms, resulting in unclear cognition of the specific content of this behaviour in practice and difficulty accurately assessing the degree and stage of adverse psychological reactions to patients. There are some problems such as the lack of pertinence and consistency in the records of psychological diagnosis and treatment, the lack of supervision and incentive feedback in the implementation process, and the difficulty in identifying and reflecting the implementation effect. A retrospective cohort study by Wang and colleagues [27] showed that a standardized trauma medical system can optimize the diagnosis and treatment process of trauma patients and shorten the treatment time. This research found that most hospitals have set up emergency trauma and orthopedic trauma units. However, in these specialized units, medical staff focused more on body or function trauma for patients. Pain management has been widely valued, but short-term or long-term psychological problems of trauma patients might be ignored. Trauma psychological care programs varied greatly in different hospitals, information exchange and sharing mechanism was rarely established among care teams, and some nurses had weak perception of providing psychological care for patients. As a nurse who worked in trauma units has the most contact with trauma patients, nurses should give full play to their ability of observation and professional treatment, set up psychological assessment and care team, build a bridge of communication between doctors and patients, promote communication and mutual assistance between different trauma centers or units, so as to play a leading role in the trauma units. Thus, managers, researchers and practitioners should strengthen cooperation and collaboration, use information technology, establish multidisciplinary collaborative, combine different classification of traumatic mechanisms, establish and improve the trauma patients’ psychological care specification process and quality supervision mechanism, to guide medical personnel to implement further, coherent, targeted psychological nursing practice.

## Conclusions

Medical staff generally thought that the content and form of psychological care were not rich enough and lacked of professionalism in the process of psychological care for trauma patients. Affected by their own and external factors, the implementers were not active enough to carry out psychological care for trauma patients. They were faced with the following problems: difficult identification of psychological characteristics and needs of different trauma patients, unclear definition and understanding of the specific content of psychological care, absent standardized psychological care procedures and norms, lack of whole-process evaluation and supervision mechanism, and unclear mechanisms of medical cooperation. Therefore, managers should enrich the content and form of psychological care training to improve the awareness and ability of psychological care of medical staff in the field of trauma, formulate reward and punishment mechanisms in the implementation process, and improve the attention and professional ability of the implementer, to stimulate their psychological care practice.

## Strengths and limitations

This study attempted to explore the experience of Chinese medical staff in psychological care of trauma patients with a qualitative method. The methodology generated four major themes that give an in-depth understanding of the promotion and hindrance factors of medical staff's cognitive experience and behavior, aiming to promote shared implementation decision making. However, the participants were only recruited from 6 hospitals in southwestern China and could not represent all medical staff working in the trauma field. Some contextual characteristics such as location, hospital size, structure, and local environment might differ from that of other healthcare settings. Therefore, the results of this study might be considered whether or not allow for the generalization to other settings. Besides, potential bias is that the lower number of male nurses included in our study, this may result in lacking of information from their point of view. Further research should be designed to explore their perceptions, barriers and facilitators of psychological care with a large and diverse sample of medical staff, particularly whilst faced on different stages, degrees and types of trauma patients, and identify methods to equip medical staff with the ability to offer psychological care is meaningful within their future multidisciplinary teams.

## Data Availability

The datasets used in this study was available from the corresponding author upon reasonable request.
